# Autoantibodies against CD80 in patients with COPD

**DOI:** 10.1038/cti.2016.57

**Published:** 2016-10-07

**Authors:** Xu Min Luo, Xin Yan Liu, Ji Hong Tang, Wei Yang, Zhen Hua Ni, Qing Ge Chen, Xiongbiao Wang

**Affiliations:** 1 Department of Respiratory Medicine, Putuo Hospital Affilated Shanghai University of Traditional Chinese Medicine, Shanghai, China

## Abstract

Chronic obstructive pulmonary disease (COPD) is an inflammation disorder and possibly an autoimmune disease. The components of the autoimmune response in the circulatory system are of considerable interest to clinicians. Because aberrations of costimulation status have been noted in COPD, the presence of autoantibodies to B7 costimulatory factor CD80 were investigated in a cohort of patients. Recombinant rs1CD80 (lacking the transmembrane domain of CD80) was used for Western blot analysis and ELISA to investigate the presence of autoantibodies in sera of patients with stable COPD and in controls without COPD. Cytokines IL-6 and IL-8 were detected using ELISA. Western blot revealed a specific band reacting to rs1CD80 by diluting sera pool of patients, which indicated the existence of autoantibodies to CD80. The serum level of anti-rs1CD80 was higher in patients with COPD than in controls(*P*=0.0185) and was positively correlated to the serum level of IL-6 (*r*=0.797, *P*<0.001) and IL-8 (*r*=0.608, *P*<0.001). There was a tendency that more higher level of anti-rs1CD80, more severe COPD stage. The existence of autoantibodies to costimulatory factor CD80 may suggest a pathogenic role of costimulatory factors in COPD.

According to GOLD2013, chronic obstructive pulmonary disease (COPD) is characterized by persistent airflow limitation that is associated with an enhanced chronic inflammatory response to noxious particles or gases. This chronic inflammatory response may induce parenchymal tissue destruction (resulting in emphysema), and disrupt normal repair and defense mechanisms (resulting in small airway fibrosis).^1^ However, it shares many clinical and pathophysiological features with autoimmune diseases.^2, 3^ Data have confirmed that peripheral T cell function is correlated with the severity of COPD.^4, 5^ Several autoantibodies in COPD patients have also been reported.^6, 7, 8^

Costimulatory factors are cell-surface proteins and play a crucial role in the immune response.^9^ CD80/CD86 and their receptors CD28/CD152 are primary costimulatory factors. Lack of CD28 induces immune anergy and lack of CD152 induces excessive immune response. Their expression affects the intensity and direction of the immune response. There are multiple forms of regulation system for expression. One form of the regulation is multiple alternative splicing of mRNA. Multiple gene products from a single gene can act as agonists or antagonists, and some possess novel functions. It has been confirmed that unstimulated peripheral blood mononuclear cells (PBMCs) express soluble forms of mRNA for CD28, CD152 and CD80, which lack transmembrane domains.^10, 11, 12^ Soluble CD28, CD152 and CD80 have also been detected in human serum.^13^ The genes may also be regulated by the autoantibody network. Many membrane proteins give rise to autoantibodies, which affect the expression and function of the proteins. There may be autoantibodies to costimulatory factors secreted as well. Autoantibodies to surface molecules on lymphocytes, also known as anti-lymphocyte antibodies (ALAs), were first described 10 years ago. The known targets of ALAs are HLA class I molecules,^14^ β2-microglobulin^15, 16^ and CD45.^17, 18^ The autoantibodies to CD152 and CD28 have been the subject of recent studies.^19, 20^

CD80 is one of the main ligand of CD28 and CD152, and there are several spliced forms of mRNA.^12^ There is abnormal expression of CD80 in COPD.^21^ It is here hypothesized that the possible presence of autoantibodies to CD80 may play a role in the inflammatory and autoimmune components of COPD. The recombinant soluble form rs1CD80, which contains intact extracellular domain, here served as an antigen for detecting the autoantibodies.^12^ The specificity and function of rs1CD80 had been reported.^12^

## Results

### Detection of autoantibodies against CD80 by immunoblot

**Figure 1:**
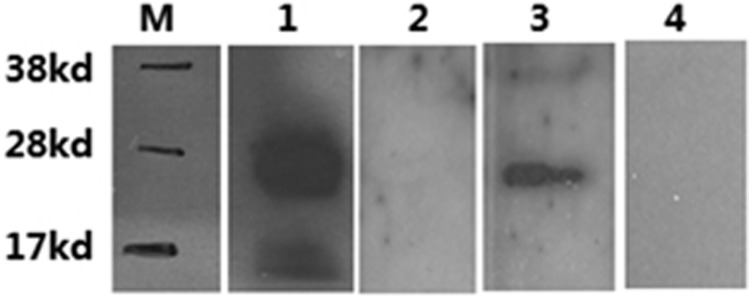
The present of autoantibodies to rs1CD80 in sera confirmed by western-blot. The band present at level of ≈30 kDa in lane 1 indicated specific binding of recombinant rs1CD80 with anti-CD80 mAb and the band in lane 3 indicated the presence of auto-antibodies in sera to rs1CD80. M: molecular marker. 1. positive control. 2. Human IgG. 3. COPD sera. 4. control sera.

Rs1CD80 was tested for binding of monoclonal antibodies against CD80 using Western blots. SDS–polyacrylamide gel electrophoresis (SDS–PAGE) and immunoblot analysis of recombinant protein showed that it was detected by monoclonal antihuman CD80 antibodies (lane 1, Figure 1) but not by polyclonal antibodies against human IgG (lane2, Figure 1). These results indicated that the protein has a distinct CD80 fragment but it did not contain residues of the Ig-molecule. This excluded any cross-reactions between circulating antibodies directed to Fc-fragment. The secondary antibodies used did not show any nonspecific binding (data not shown). For detection of autoantibodies against CD80 with this method, sera from a pool of six male, aged 60–70, no-smoking COPD patients were tested (lane 3, Figure 1) and from a pool of six matched healthy individuals (lane 4, Figure 1). CD80 autoantibodies presented as a distinct band in COPD serum pool.

### Presence of autoantibodies to rs1CD80 in sera from patients with COPD

**Figure 2:**
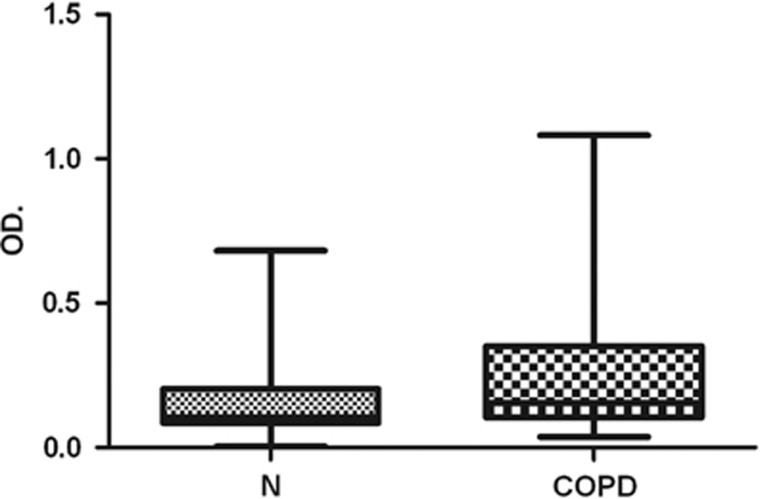
The levels of rs1CD80 autoantibodies in sera from patients with COPD and control subjects, expressed as OD units. The boxes represent the means +s.d. OD: optical density

One hundred fifty matched sera from COPD patient and controls were test for autoantibodies to rs1CD80. Figure 2 shows the ELISA data of autoantibodies after correction for background signals. The OD value for anti-rs1CD80 autoantibodies were significantly higher in COPD patients than in controls (*P*=0.0185). There was no difference in the total IgG concentration between the subjects with higher or lower OD values (data not shown).

### Relationship between rs1CD80 autoantibodies and inflammation markers

**Figure 3:**
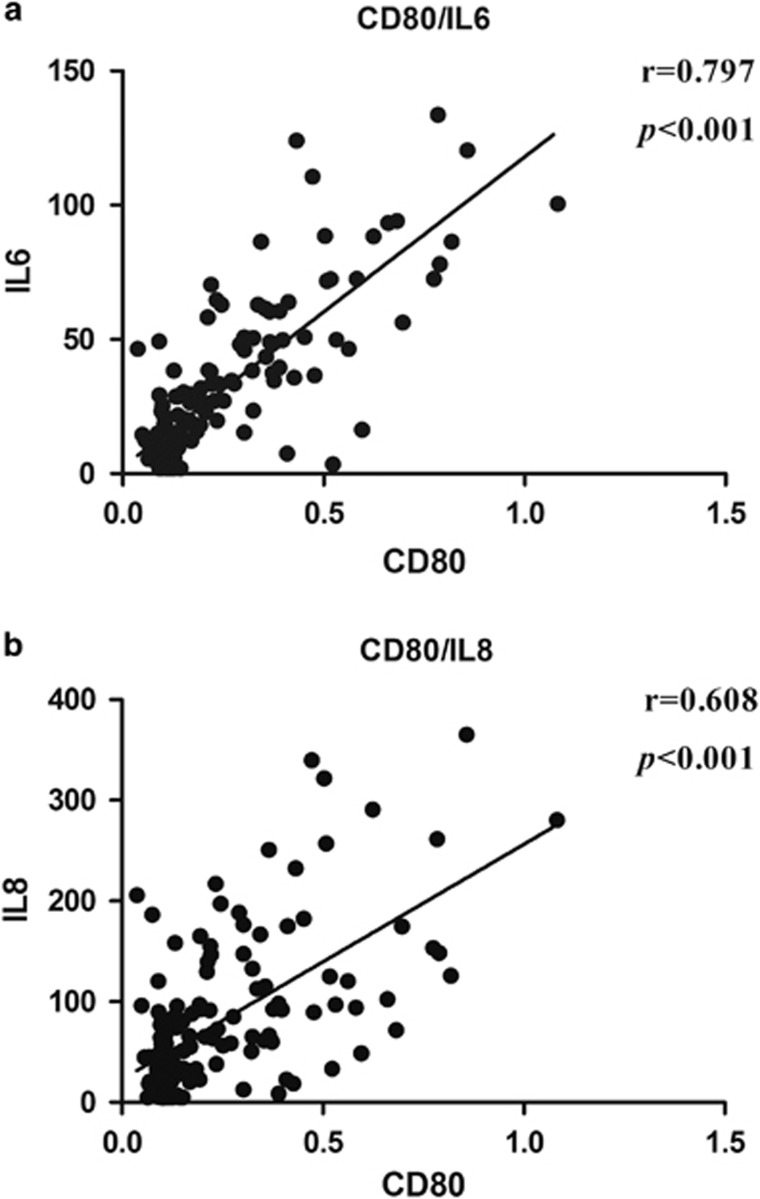
The relationship of the serum of IL-6/IL-8 with anti-CD80 autoantibodies. (**a**) The level of anti-CD80 is positively related with IL-6 and (**b**) the level of anti-CD80 is positively related with IL-8.

The results of cytokine measurement were consistent with previous reports that the serum levels of IL-6 and IL-8 were higher in COPD patients.^22, 23^ In this study, the mean value of COPD group is 46.914±31.756 pg ml^−1^ for IL-6 and 80.283±62.641 pg ml^−1^ for IL-8 in COPD group, respectively. Results showed that the serum levels of IL-6 and IL-8 in COPD were closely associated with the titers of auto-antibodies to rs1CD80 (*r*=0.797, *P<*0.001 and *r*=0.608, *P<*0.001, respectively) (Figures 3a and b)

### Relationship between rs1CD80 autoantibodies and clinical parameters

**Figure 4:**
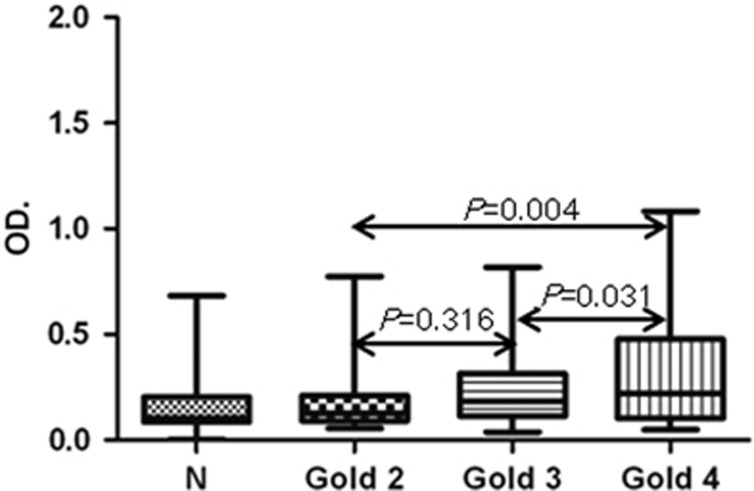
The levels of anti-CD80 autoantibodies in subgroups of COPD patients. The results expressed as means+s.d. GOLDII, III and IV represent different stages of COPD; N, control.

Different stages of COPD tended to show distinct differences in anti-rs1CD80 autoantibody levels (Figure 4). More severe patients appeared more higher level of anti-rs1CD80(GOLD2:GOLD3, *P*=0.316; GOLD2:GOLD4, *P*=0.004; GOLD3:GOLD4, *P*=0.031). There was no difference of age between stages and no significant relationship was detected between smoking history and the levels of autoantibodies rs1CD80 (data not shown).

## Discussion

Recombinant s1CD80 was prepared and the presence of autoantibodies against it was investigated in sera obtained from patients with COPD and controls without COPD. This is the first study of the existence of autoantibodies to costimulatory factors in COPD and the existence of CD80 autoantibodies in humans. In this study, we could not exclude the effect of inhaled corticosteroid on the level of CD80 autoantibodies though the dose is quite low(0.5 mg inhaled per day and about 0.1 mg intake in the lung). As corticosteroid treatment usually plays inhibitory role in producing antibodies, low dose of corticosteroid may affect the level of auto-antibodies, but the effect may be quite weak and lead to low-estimate.

Previous related studies have suggested that costimulatory molecule autoantibodies are present in inflammatory diseases at higher frequencies than healthy controls. One of these studies showed CD28 autoantibody to be present in 53 of 196 (27.04%) patients with atopic diseases and 8 of 72 (11.1%) healthy controls. Another study showed CD152 autoantibody to be absent from healthy individuals but present at high concentrations in patients with several autoimmune diseases.^24^ The present of anti-lymphocyte autoantibodies has been demonstrated in systemic lupus erythematosus,^25^ HIV infection^26^ and rheumatoid arthritis.^27^ It is here confirmed the present of another autoantibody to CD80.

COPD is an inflammatory disease and its pathogenesis involves an acquired immune response to newly created or altered epitopes.^28^ IgG autoantibodies with avidity for pulmonary epithelium are prevalent in COPD patients.^7^ In the present study, IgG autoantibodies to costimulatory factor were found in COPD patients. The serum level of CD80 autoantibodies was significantly higher in COPD patients than in controls, which suggests that it might be a risk factor for the pathogenesis. It is here proposed that the autoantibodies are not specific to COPD or autoimmune diseases, but they should be indicative of an autoimmune status.

Autoantibody production depends on antigen presentation and tolerance of autoantigens. A relative higher level of anti-CD80 autoantibodies in COPD may suggest the disorder of antigen-present-cells, antigen-present-cells may express more membrane form or soluble form of CD80. Freeman CM found that CD80 expression on dendritic cells was increased with disease progression in COPD.^21^ This evidence supports the current conclusion that the level of anti-CD80 autoantibodies was associated with COPD stage.

The function of the autoantibodies was found to vary, mostly depending on the identity of the antigen. ALAs may be able to modulate lymphocyte function by binding to their target molecules on the cell surface. The occurrence of ALAs may be associated with disease activity in systemic lupus erythematosus.^29^ ALAs can damage the function of T cells, B cells and monocytes.^30, 31, 32^ The autoantibodies against costimulatory factor may act not only as ALAs to lyse activated T cells and antigen-present-cells via antibody-dependent cell-mediated cytotoxicity, but also as modulators of the immune response via blocking or activating costimulatory signaling. Matsui's results indicate that anti-CD152 autoantibodies can block inhibitory signals mediated by CD152 molecules on T cells and so render the T-cell response more pronounced.^19^ Autoantibodies against CD28 were found stimulate T cells and overcome the CD152-Ig-induced anergy of T cells in an mixed lymphocyte reaction.^20^ The function of autoantibodied to CD80 need to be clarified.

Autoantibodies to CD80 might be associated with COPD. Aberrant expression of CD80 in COPD may induce the high level of anit-CD80. Beside the evidence of increased expression of CD80 on DC,^33^ possible abnormal expression of membrane form on other immune cells and alternation expression of no membrane form in the immune cells airway epithelial cells may provide more antigens for developing autoantibodies. The results of the current study showed that the level of anti-CD80 autoantibodies to be closely correlated with inflammation markers IL-6 and IL-8. The more severe the COPD, the higher the level of anti-CD80 autoantibodies. So, anti-CD80 autoantibodies may therefore affect the intensity of immune response through costimulatory signal to modulate the level of inflammation. Further studies on patients at the early stage of COPD are needed.

The production of autoantibodies is not a single independent event in the immune system. Rather, it involves a complex network. The autoantibodies to costimulatory factors were found to interfere with the normal receptor–ligand interactions and modulate the surface level of costimulatory factors, leading to abnormal function of the costimulatory system. The high level of autoantibodies against CD80 suggests that an aberrant immune response maybe involved in the pathogenesis of COPD. Further studies on the level of expression of cofactors and the presence of autoantibodies against other costimulatory factors should be interesting.

## Methods

### Generation of His-tagged Recombinant s1CD80

The method for generation of rs1CD80 is the same as described in our previous study.^12^ Briefly, the s1CD80, lack transmembrane domain, was expressed in *Escherichia coli*. The protein was purified by the Ni-NTA His.Bind Resins (Novagen, Darmstadt, Germany) under denaturing conditions and renaturation was executed.

### Western-blot analysis

Rs1CD80 was run via SDS–PAGE(12%gel) at reducing conditions using the NuPage gel system (Invitrogen, Shanghai, China) and transfered onto a polyvinyl difluoride membranes (Amersham Biosciences, China). After blocking with 5% skimmed milk for 60 min at room temperature and washing in TBS (three times) the membranes were cut into strips. For control experiments the strips were incubated with monoclonal mouse antihuman CD80 (R&D Systems, China) or polyclonal rabbit antihuman IgG(R&D Systems), diluted 1: 1000 in TBS with 1%BSA and 0.2%Tween-20 for 1 h. Biotinylated polyclonal goat antimouse IgG or goat antirabbit IgG (Dakocytomation, Denmark) was applied as secondary antibodies. For detection of CD80 autoantibodies, the strips were incubated for 1 h at room temperature in 1:10 diluted sera. After washing three times, the second antibody (biotinylated polyclonal goat antihuman IgG, Dakocytomation) was added and the membranes were incubated for 1 h at room temperature. The detection using avidin-HRP conjugate (Zymed, San Francisco, CA, USA). ECL-Plus (Amersham Biosciences) was used as substrate. The bands were visualized after exposed to hyperfilms (Amersham Biosciences).

### Development of ELISA to detect autoantibodies against the rs1CD80

The recombinant protein (1 mg l^−1^) was coated on the ELISA plates (NUNC-Immuno Plate; Nalge Nunc International, Denmark) and diluted in PBS overnight at 4 °C. The plates were washed twice with PBS and 0.05% Tween-20 and blocked by 0.5% BSA in PBS. The sera were diluted 10 times and loaded into the wells. The plates were incubated for 1 h at 37 °C and washed four times. The HRP(Horseradish Peroxidase) conjugate was added (anti-human IgG/HRP) (Dakocytomation), 1:10 000 dilution in reagent buffer (Tris-buffered saline, 0.05% Tween-20). The plates were incubated for 1 h at room temperature. A final washing step (four times) was followed by addition of 3,3',5,5'-tetramethylbenzidine (Sigma-Aldrich, St Louis, MO, USA). After 15 min incubation at room temperature, the reaction was stopped using 1.8 N H_2_SO_4_. Optical density(OD) value was read using an ELISA reader at 450 nm, with correction values at 540 nm. The titer of the autoantibodies was calculated based on the OD value.

### Quantification of IL-6, IL-8 by ELISA

The sera of patients were also used for detection of IL-6, IL-8 by ELISA kits (R&D systems) according to instructions of manufactory.

### Study population

The control serum samples were collected from 150 matched (age, gender and smoking) individuals who lived in the western part of Shanghai during healthy examination. Total 150 patients with stable COPD were in follow-up by Shanghai Putuo Hospital in 2011–2013, who were lived in the same area as the controls. The diagnosis of COPD was followed with GOLD2012.^1^ According to GOLD classification of airflow limitation as determined by spirometry, 42 patients were moderate(50% ≤FEV_1_<80% predicted), 56 patients were severe (30% ≤FEV_1_<50% predicted) and 52 were very severe COPD(FEV_1_<30% predicted). The sera were collected in stable stage and saved in −80 °C. Moderate patients were treated with shorter-acting or long-acting β2-agonists, anticholinergics. The addition of inhaled glucocorticosteroids to bronchodilator treatment was applied in GOLD III and GOLD IV COPD patients (severe and very severe). No systemic glucocorticosteriods had been adopted in all patients for two months before sera collection. Patients were excluded if they had a history of respiratory infection within the last two months, history of any other significant concomitant pulmonary or extrapulmonary disease or treatment with anti-inflammatory agents or antihistamines within 6 days. The former smokers had a smoking abstinence of >15 years and a smoking history of 20-pack years. The current smokers had a smoking history of >20 pack-years. The characteristics of participants are listed in Table 1. The pools were mixed sera from six individuals of COPD and control respectively. The Shanghai University of Traditional Chinese Medicine Ethics Committee for Scientific Research approved the study protocol, and written consent was obtained from all participants.

### Statistical analysis

Comparisons between the values in patients and controls were made using the non-parametric Mann–Whitney *U* rank sum analysis. Correlations between different assays were made using the Spearman rank correlation. Spearman correlations were calculated using GraphPad Prism software. *P*<0.05 was considered to be statistically significant.

## Figures and Tables

**Table 1:** Characteristics of the study subjects

	*Control*	*COPD*
Number	150	150
Gender (F/M)	98/52	98/52
Age (years), Mean (s.d.)	69.4±6.57	68.1±6.82
Smoking status		
Never	53	58
former	80 (means18.72PY)	77 (means21.31PY)
current	17 (means40.43PY)	15 (means42.8PY)
Chronic condition		
Hypertention	22	19
Diabetes	18	18
Stroke	2	0
Arthritis	3	2
FEV1%pred (Means±s.d.)	83.24±16.93	42.16±27.64
50% ≤FEV_1_<80%predicted (*n*)		42
30% ≤FEV_1_<50% predicted (*n*)		56
FEV_1_<30% predicted (*n*)		52

Abbreviation: PY, consumption packs × year.
